# 11β-Hydroxysteroid Dehydrogenase Type 1 (11β-HSD1) Inhibitors Still Improve Metabolic Phenotype in Male 11β-HSD1 Knockout Mice Suggesting Off-Target Mechanisms

**DOI:** 10.1210/en.2013-1613

**Published:** 2013-10-29

**Authors:** Erika Harno, Elizabeth C. Cottrell, Alice Yu, Joanne DeSchoolmeester, Pablo Morentin Gutierrez, Mark Denn, John G. Swales, Fred W. Goldberg, Mohammad Bohlooly-Y, Harriet Andersén, Martin J. Wild, Andrew V. Turnbull, Brendan Leighton, Anne White

**Affiliations:** Neuroscience Research Group (E.H., A.W.), Faculty of Life Sciences, University of Manchester, Manchester M13 9PT United Kingdom; Centre for Endocrinology and Diabetes (E.C.C., A.W.), Faculty of Medical and Human Sciences, University of Manchester, Manchester M13 9PT United Kingdom; CVGI iMed (A.Y., J.D., F.W.G., A.V.T., B.L.) and DMPK (P.M.G., M.D., J.G.S., M.J.W.), AstraZeneca, Alderley Park, SK10 4TG, United Kingdom; and Transgenic RAD (M.B., H.A.), Discovery Sciences, AstraZeneca, Mölndal S-431 83, Sweden

## Abstract

The enzyme 11β-hydroxysteroid dehydrogenase type 1 (11β-HSD1) is a target for novel type 2 diabetes and obesity therapies based on the premise that lowering of tissue glucocorticoids will have positive effects on body weight, glycemic control, and insulin sensitivity. An 11β-HSD1 inhibitor (compound C) inhibited liver 11β-HSD1 by >90% but led to only small improvements in metabolic parameters in high-fat diet (HFD)–fed male C57BL/6J mice. A 4-fold higher concentration produced similar enzyme inhibition but, in addition, reduced body weight (17%), food intake (28%), and glucose (22%). We hypothesized that at the higher doses compound C might be accessing the brain. However, when we developed male brain-specific 11β-HSD1 knockout mice and fed them the HFD, they had body weight and fat pad mass and glucose and insulin responses similar to those of HFD-fed Nestin-*Cre* controls. We then found that administration of compound C to male global 11β-HSD1 knockout mice elicited improvements in metabolic parameters, suggesting “off-target” mechanisms. Based on the patent literature, we synthesized another 11β-HSD1 inhibitor (MK-0916) from a different chemical series and showed that it too had similar off-target body weight and food intake effects at high doses. In summary, a significant component of the beneficial metabolic effects of these 11β-HSD1 inhibitors occurs via 11β-HSD1–independent pathways, and only limited efficacy is achievable from selective 11β-HSD1 inhibition. These data challenge the concept that inhibition of 11β-HSD1 is likely to produce a “step-change” treatment for diabetes and/or obesity.

As rates of metabolic syndrome and its component conditions of obesity, type 2 diabetes, and hypertension continue to rise (1), there is an increasing need to find improved therapies to treat these disorders. Glucocorticoids are implicated as causal in promoting both obesity and insulin resistance, the latter of which is a key stage in the progression to type 2 diabetes. Exposure to excess glucocorticoids, as occurs in Cushing syndrome, drives hyperphagia, body weight gain, hyperlipidemia, and insulin resistance.

Circulating glucocorticoids are derived at least in part by intracellular regeneration of active steroids (cortisol in humans and corticosterone in rodents) from inactive metabolites (cortisone/11-dehydrocorticosterone) by the enzyme 11β-hydroxysteroid dehydrogenase type 1 (11β-HSD1). In obese human subjects, circulating cortisol levels do not correlate with body mass index or glucose and insulin concentrations (2) because there is increased cortisol clearance (3). However, increased tissue 11β-HSD1 expression and activity have been demonstrated, notably in metabolic tissues including liver and adipose tissue (4–7). This finding has led to the widely held belief that elevated 11β-HSD1 in tissues may be contributing to metabolic disease (8, 9).

Several elegant studies have highlighted the role of 11β-HSD1 in metabolic syndrome. Mice with global 11β-HSD1 knockout (GKO) have lower body weight when fed a high-fat diet (HFD), less visceral fat, and lower fasting glucose, accompanied by improved glucose tolerance (10, 11). Conversely, overexpression of 11β-HSD1 in adipose tissue of mice causes hyperphagia and visceral obesity, and when fed an HFD, these mice exhibit insulin-resistant diabetes (12). This defining study provided some of the first evidence suggesting a causative link between elevated adipose 11β-HSD1 levels and insulin resistance.

Evidence from these studies in knockout and transgenic mice together with studies in humans suggested that decreasing cortisol by inhibition of 11β-HSD1 would be an attractive target for new therapeutic agents. As a result many pharmaceutical and biotechnology companies and some academic groups set up programs to develop 11β-HSD1 inhibitors as a potential therapy for type 2 diabetes. In preclinical studies with C57BL/6J mice fed an HFD, the beneficial effects of 11β-HSD1 inhibition were observed, including reduced body weight, food intake, and fasting glucose and insulin levels (13–17). More recently, phase IIb clinical trials with 11β-HSD1 inhibitors resulted in improved glucose homeostasis and decreased body weight in type 2 diabetic subjects (18, 19). However, only high doses of 11β-HSD1 inhibitors (and very high levels of 11β-HSD1 inhibition) improve glycemic control in humans and even then they only have modest effects (18, 19).

Another inhibitor of 11β-HSD1 (compound C discovered by AstraZeneca) is highly effective in reducing enzyme activity both in vitro and in mouse studies. However, significant beneficial effects on the metabolic phenotype were only seen when high doses of the inhibitor were used. We therefore explored whether these compounds were having their beneficial effects by central nervous system (CNS) inhibition of 11β-HSD1, which required the higher doses of inhibitor to access the CNS or whether administration of high doses of the inhibitor caused “off-target” effects. Our data suggest that a significant component of the beneficial effects of 11β-HSD1 inhibitor administration on body weight and glycemic control occurs via 11β-HSD1–independent mechanisms and call into question the validity of this enzyme as a drug target for the treatment of type 2 diabetes and obesity.

## Materials and Methods

### Animals and genotyping

The *HSD11B1* gene–targeting vector was prepared from a 129/Sv BAC clone (ResGen; Invitrogen). DNA fragments of 5.2, 1.1, and 2.6 kb (for 5′ homology, deletion, and 3′ homology regions, respectively, were cloned into a modified loxP floxed PGKneo plasmid, linearized, and electroporated into R1 mouse embryonic stem (ES) cells. The deletion fragment includes exon 2. Candidate ES cell clones were screened by PCR and confirmed by Southern blotting to identify homologous recombination. Targeted ES cells were injected into C57BL/6J blastocysts, and embryos were implanted into pseudopregnant B6CBA female mice. Chimeric animals were mated with C57BL/6 mice to produce agouti heterozygous animals (F1).

For generation of GKO mice, heterozygous F1 males were bred with Rosa26-*Cre* knock-in mice (kindly provided by Dr. Philippe Soriano [20] and backcrossed onto a C57BL/6 background at AstraZeneca) to delete the loxP floxed region including exon 2 and the neo cassette, and GKO mice for studies were obtained by intercrossing. For generating 11β-HSD1 conditional (floxed) mice, the heterozygous F1 mice were bred with EIIa-*Cre* knock-in mice (C57BL/6 background) to delete the loxP flanked PGKneo cassette.

Offspring were genotyped at weaning by PCR using specific primers to detect the wild-type allele (1.6-kb fragment) and the null allele (0.6-kb fragment), respectively (forward, CAGGTGAGTACCACTGTGTCTCATTT; reverse, AGTCCGCCTGCAAAGAGATAGATG). For conditional mice, the primers used detected the wild-type allele (315 bp) or the floxed allele (440 bp) (forward, GATCTGCCCACCTTTGCCTTCT; reverse, CCATGAGCTTTCCCGCCTTGACA, respectively).

*Cre*-loxP technology was used to generate mice with CNS-specific disruption of the *HSD11B1* gene (brain knockout [BKO]). Male mice heterozygous for the conditional *HSD11B1* allele and *Cre* recombinase under the control of the rat nestin promoter (Charles River Laboratories) were mated with female homozygous conditional *HSD11B1* mice. The resulting *Cre*-positive 11β-HSD1^lox/lox^ (BKO) and *Cre*-negative 11β-HSD1^lox/lox^ (wild-type [WT^f/f^]) offspring were determined by genotyping at weaning. The primers used were GCTACTTCTTTTCAACCCCTAAAA (forward) and CTGGATAGTTTTTACTGCCAGACC (reverse), giving a band size of 420-bp in BKO mice and no band in WT littermates.

C57BL/6J mice used for inhibitor characterization studies were bred in-house (AstraZeneca). All work was performed in accordance with the UK Home Office Animals (Scientific Procedures) Act 1986 and approved by the local ethics review committee.

### Compound details

Compound C (2-[(2*S*,6*R*)-2,6-dimethylmorpholin-4-yl]-*N*-(5-hydroxy-2-adamantyl)-4-[(2*R*)-oxolan-2-yl]pyrimidine-5-carboxamide) has an IC_50_ potency of 0.07 μM in the mouse as measured by homogeneous time-resolved fluorescence. It is in the same chemical series as previously published compounds (21, 22). MK-0916 (3-[1-(4-chlorophenyl)-3-fluorocyclobutyl]-4,5-dicyclopropyl-1,2,4-triazole) was synthesized from the patent literature, and its effects have been characterized previously (Patents WO2003104207, WO2005073200, and WO2007038452 [23, 24].

### Experimental design

Mice were fed standard chow (RM1, Special Diet Services Ltd) or the HFD (60% kcal from fat, D12492; Research Diets) ad libitum for up to 30 weeks to induce obesity. During this period and throughout the studies, the mice were handled on a regular basis and were therefore acclimatized to handling in advance of any procedures. Small molecule 11β-HSD1 inhibitors were administered to the mice via compound in diet for up to 25 days. For inhibitor studies, food intake was measured before the treatment phase to calculate the amount of compound required for diet incorporation and in all studies during the treatment phase to examine changes in food intake with compound or gene deletion. For determination of fasting glucose, mice were fasted for up to 18 hours before tail venesection, and glucose was measured using an ACCU-CHEK glucometer (Roche). At the end of the study, animals were euthanized under CO_2_/O_2_, and death was confirmed by cervical dislocation. Mesenteric, inguinal (as a measure of subcutaneous), and epididymal adipose tissues were excised and weighed. Adipose tissues, liver, and brain were snap-frozen for subsequent mRNA analysis and enzyme activity measurements.

Compound levels were measured in plasma and brain after extraction in acetonitrile by liquid chromatography–tandem mass spectrometry (Thermo TSQ Vantage; Thermo Scientific) to determine exposure levels. Free plasma levels of compounds were calculated based on the measured compound concentrations in plasma and then were corrected using a constant free fraction (fu) for each compound. These were generated in-house in vitro using previously published methodology (25). Free drug levels in brain were calculated using a constant free fraction in brain (fu_b_) obtained with methodology similar to that for fu. For compound C, fu and fu_b_ were 0.43 and 0.27, respectively. For compound MK-0916, fu and fu_b_ were 0.08 and 0.06, respectively. In the HFD experiments, brain concentrations were predicted at the different time points based on measured free compound concentrations in plasma, and a constant [free]_brain_/[free]_plasma_ ratio for each compound was determined in previous experiments (data not shown).

To represent the level of cover for each compound, the resulting free compound concentrations in plasma and brain were divided by their respective in vitro IC_50_ values. IC_50_ values used were 0.07 and 0.03 μM for compound C and MK-0916, respectively.

### In vitro potency (IC_50_) assessment

The conversion of cortisone to cortisol by 11β-HSD1 oxoreductase was measured using a cortisol competitive homogeneous time-resolved fluorescence assay (Cisbio International). The assay incubation was performed in black 384-well plates (Matrix) consisting of cortisone (160 nM), glucose 6-phosphate (1 mM), NADPH (100 μM), glucose-6-phosphate dehydrogenase (12.5 μg/mL), EDTA (1 mM), assay buffer (K_2_HPO_4_/KH_2_PO_4_, 100 mM), pH 7.5, with recombinant 11β-HSD1 (1.5 μg/mL) plus test compound in a total volume of 20 μL. The assay was incubated for 25 minutes at 37°C, and the reaction was stopped by the addition of 10 μL of 0.5 mM glycyrrhetinic acid plus cortisol-XL665. Anticortisol cryptate was then added to the plates and incubated for 2 hours at room temperature. Fluorescence was measured and the 665 nm to 620 nm ratio was calculated using an EnVision plate reader. These data were used to calculate IC_50_ values (Origin 7.5).

### 11β-HSD1 activity assay

Enzyme activity (knockout studies) and target engagement (compound studies) were determined ex vivo by measuring conversion of [^3^H]cortisone to [^3^H]cortisol. Tissues were taken from knockout mice or from mice treated with 11β-HSD1 inhibitors as appropriate. In brief, tissues were incubated for 10 minutes (liver), 45 minutes (brain), or 60 minutes (adipose tissue) in the presence of [^3^H]cortisone (20 nmol/L, 1 μCi/mL, specific activity 1.97 GBq/mmol; PerkinElmer). Radiolabeled steroids were extracted using ethyl acetate and separated using reverse-phase HPLC.

### Quantitative mRNA analysis

Total RNA was extracted from brain, hypothalamus, liver, and epididymal adipose tissue using either TRIzol reagent (brain and adipose tissues; Invitrogen) or an RNeasy mini kit (hypothalamus and liver; Qiagen), according to the manufacturer's instructions. Contaminating genomic DNA was removed using a Turbo DNAse kit (Ambion). RNA quantity and integrity were determined (ND1000; NanoDrop Technologies), before 1-step quantitative RT-PCR was performed using a TaqMan RNA-to-C_T_ 1-Step Kit (Applied Biosystems) for measurement of *HSD11B1* mRNA expression (Applied Biosystems). Specific TaqMan probes were used to amplify a region of exon 2 and 3 of the *HSD11B1* gene (Mm01313990_m1), and values were normalized to levels of hypoxanthine-guanine phosphoribosyltransferase (*HPRT*; Mm00446968_m1) using standard curve analysis.

### Oral glucose tolerance test (OGTT)

Mice were fasted for 16 hours before baseline blood glucose was measured (ACCU-CHEK glucometer), and blood samples were collected for insulin measurement (ELISA; Crystal Chem) by tail venesection. Mice were administered 10 mL/kg of 20% glucose by oral gavage (0 minutes), and glucose was measured at 20, 40, 60, and 90 minutes. Blood samples were collected for insulin measurements at 20 and 40 minutes.

### Assays of circulating analytes

Tail venesection blood samples from free-running mice were taken at the previously determined peak and nadir of corticosterone. Corticosterone was measured in these samples by a commercially available kit (Cayman Chemical). Terminal plasma samples from fed or overnight fasted mice were used to measure plasma lipids and leptin. Nonesterified fatty acids (NEFAs) (Wako Chemicals), triglycerides (Sigma), and leptin (Millipore) were measured by commercially available kits.

### Dual-energy x-ray absorptiometry (DEXA) scan

Mice were anesthetized using halothane and were scanned in a Lunar PIXImus DEXA scanner (GE Healthcare). The associated software calculated the percentage fat based on the scan.

### Statistics

Data are expressed as means ± SEM. Statistical analysis was performed using Prism 5 (GraphPad Software). The unpaired Student *t* test or one-way or two-way ANOVAs with Bonferroni post hoc tests were used as appropriate. *HSD11B1* gene expression data were not normally distributed and so were analyzed by the Mann-Whitney *U* test. *P* < .05 was considered significant.

## Results

### Inhibition of 11β-HSD1 improves the metabolic phenotype of HFD-fed mice, but only at very high doses

**Figure 1.**
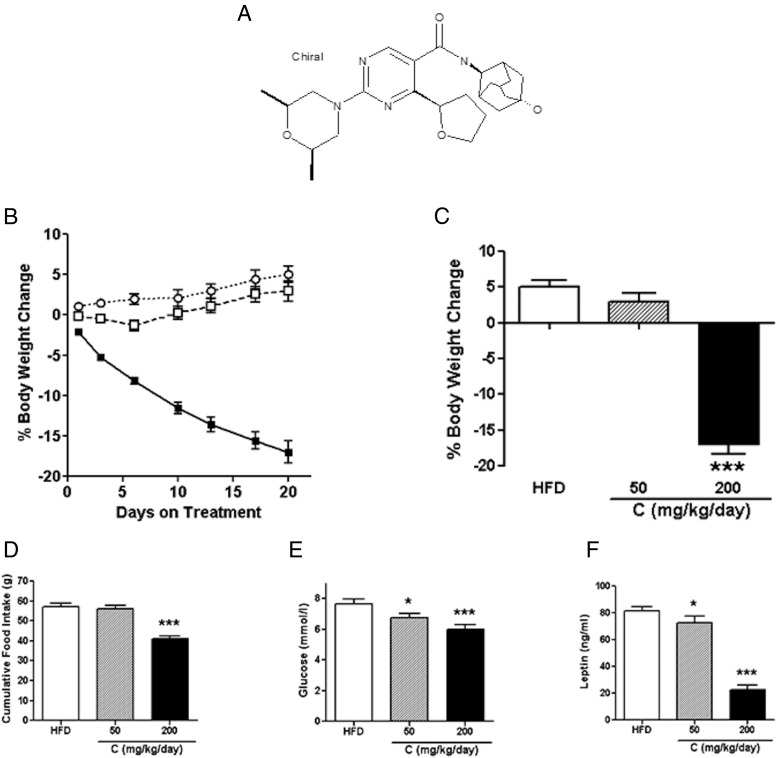
The small molecule 11β-HSD1 inhibitor, compound C, lowers body weight, food intake, and glucose in obese mice. A, Structure of compound C. B, Percentage body weight change over 20 days of treatment. ○, vehicle mice; □, 50 mg/kg/d compound C; ■, 200 mg/kg/d compound C. C, Percentage body weight change on day 20. D, Cumulative food intake over the first 20 days of treatment. E, Fasting glucose levels on treatment day 21. F, Fed leptin levels in terminal plasma taken on treatment day 25. Data are expressed as means ± SEM; n = 7 to 10. *, *P* < .05; ***, *P* < .001.

Compound C is a novel pyrimidine 11β-HSD1 inhibitor (Figure 1A), related to other compounds in the same chemical series, which have been described previously (21, 22). This 11β-HSD1 inhibitor has an IC_50_ of 0.07 μM with a plasma free fraction of 0.43. Mice fed the HFD for up to 30 weeks to induce obesity were given compound C at 50 mg/kg/d in the diet for 20 days. Steady-state levels of unbound compound in plasma were 44 to 102 times greater than the IC_50_ potency (Supplemental Figure 1B published on The Endocrine Society's Journals Online web site at http://end.endojournals.org.) and resulted in a >90% reduction in 11β-HSD1 enzyme activity in liver (Supplemental Figure 1A). This dose of the compound did not significantly lower body weight (Figure 1, B and C) or food intake (Figure 1D), although the mice tended to gain less weight than the control mice fed the HFD. However, there was a small, but significant, reduction in fasting glucose (Figure 1E) and leptin (Figure 1F) levels compared with those in the controls.

Using a higher dose of the compound (200 mg/kg/d), we achieved a steady-state unbound compound level in plasma that was 299 to 588 times greater than the IC_50_ potency (Supplemental Figure 1B). This higher dose of the compound again inhibited 11β-HSD1 by >90% and did not raise the circulating corticosterone level (data not shown). However, it reduced body weight (by 17%) (Figure 1, B and C) and food intake (Figure 1D). Fasting glucose (Figure 1E) and circulating leptin (Figure 1F) levels were also reduced in treated compared with untreated HFD-fed mice, and leptin levels were lower than those with the lower dose of the compound as well. Taken together, these data indicate that compound C has positive effects on the metabolic phenotype in obese mice, albeit only at a very high dose.

### Brain 11β-HSD1 deletion does not reduce body weight or food intake

**Figure 2.**
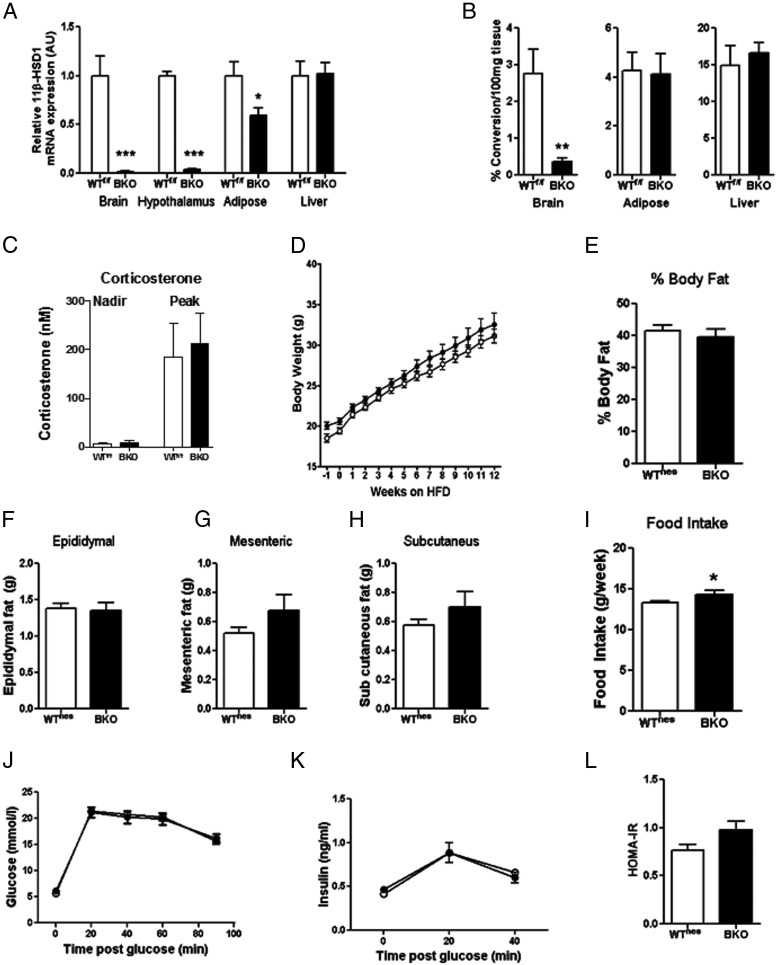
BKO mice have no reduction in body weight, fat mass, food intake, or glucose tolerance. A, 11β-HSD1 mRNA expression in brain, hypothalamus, epididymal adipose, and liver tissues. B, Enzyme activity in brain, epididymal adipose tissue, and liver in BKO and WT^f/f^ mice. C, Circulating corticosterone concentrations in BKO and WT^f/f^ mice after 18 weeks of HFD feeding. D, Body weight over 12 weeks of HFD feeding in BKO (■) and WT^nes^ (□) mice. E, Percentage body fat measured by DEXA scan after 12 weeks of HFD feeding. F–H, Epididymal (F), mesenteric (G), and subcutaneous (H) fat pad masses after 14 weeks of HFD feeding. I, Average food intake (grams per week) over 12 weeks of HFD feeding. J and K, Glucose (J) and insulin (K) excursion during an OGTT after 13 weeks of HFD feeding. L, HOMA-IR after 13 weeks of HFD feeding. Data are expressed as means ± SEM; n = 6 to 16. *, *P* < .05; **, *P* < .01; ***, *P* < .001 vs the appropriate WT strain.

Given that our selected compound inhibited liver 11β-HSD1 maximally at both doses tested but only reduced body weight and food intake at the higher dose, we considered whether the higher dose was acting in the CNS. Glucocorticoids are known to have an adverse effect on energy balance, and administration of glucocorticoids directly into the brain can both stimulate food intake (26) and inhibit peripheral glucose uptake and insulin sensitivity (27, 28). Furthermore, 11β-HSD1 is expressed within the hypothalamus (Figure 2A and Ref. 10), a key brain region mediating these effects of central glucocorticoids. We therefore examined whether the compound could penetrate the brain and found that free inhibitor concentrations in brain tissue were approximately 11 and 67 times the IC_50_ at the low and high dose, respectively (Supplemental Figure 1C). These findings support the hypothesis that our 11β-HSD1 inhibitor might be acting in the CNS. To determine the contribution of CNS 11β-HSD1 inhibition directly, we generated a CNS-specific knockout mouse by crossing mice with exon 2 of the *HSD11B1* gene flanked by loxP sites (floxed mice) with a Nestin-*Cre* strain. BKO mice had a >87% reduction in enzyme activity and a >98% reduction in mRNA expression in the brain (Figure 2, A and B). No effects of gene deletion were seen in the liver, but there was a 50% reduction in mRNA expression in epididymal adipose tissue, although this did not lead to a reduction in 11β-HSD1 enzyme activity (Figure 2, A and B), a more physiologically relevant measure of function. Deletion of 11β-HSD1 specifically in the brain did not alter circulating corticosterone concentrations (Figure 2C). In the course of our phenotyping studies, we determined that Nestin-*Cre* mice (designated WT^nes^) exhibit an intrinsic metabolic phenotype (Supplemental Figure 2 and Refs. 29, 30), and consequently all metabolic studies were performed using Nestin-*Cre* mice as controls.

Both BKO and WT^nes^ mice gained weight similarly when fed the HFD (Figure 2D), and body fat content was not different between the genotypes at the end of 12 weeks of HFD feeding, as measured using DEXA (Figure 2E). Glucose metabolism and insulin sensitivity were also not improved in BKO mice (Figure 2, J–L), and both genotypes had comparable epididymal, mesenteric, and inguinal fat pad weights (Figure 2, F–H). Food intake was increased in BKO mice compared with that in WT^nes^ mice (Figure 2I) but was significantly lower (∼15%) in both these genotypes than in WT^f/f^ and C57BL/6J mice (Supplemental Figure 2C and data not shown).

### GKO mice on a C57BL/6J background have no improvement in metabolic parameters

**Figure 3.**
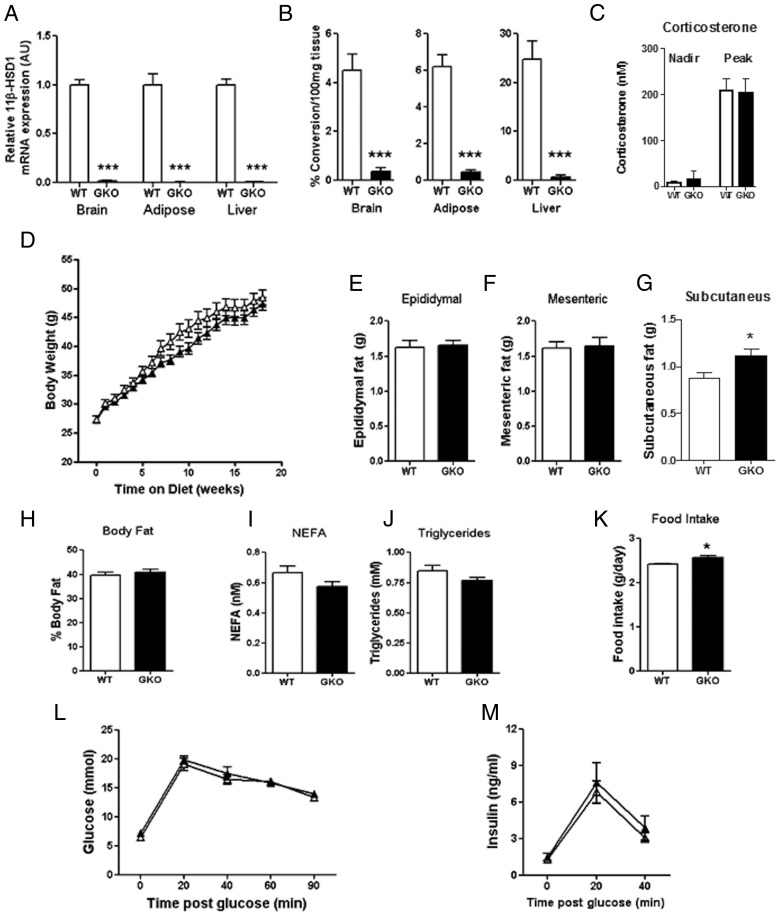
GKO mice do not have an improved metabolic phenotype compared with WT controls. A and B, 11β-HSD1 mRNA expression (A) and enzyme activity (B) in brain, epididymal adipose tissue, and liver in GKO and WT mice. C, Circulating corticosterone after 18 weeks of HFD feeding. D, Body weight over 18 weeks of HFD feeding. E–G, Epididymal (E), mesenteric (F), and subcutaneous (G) fat pad weights after 12 weeks of HFD feeding. H Percentage body fat measured by DEXA scan after 12 weeks of HFD feeding. I and J, Plasma NEFA (I) and plasma triglyceride (J) levels in fasted mice after 20 weeks of HFD feeding. K, Average food intake (grams per day) over 2 weeks in HFD-fed mice. L and M, Glucose (L) and insulin excursion during an OGTT after 13 weeks of HFD feeding. ▵, WT mice; ▴, GKO mice. Data are expressed as means ± SEM; n = 6 to 12. *, *P* < .05; ***, *P* < .001 vs WT.

Data obtained from BKO mice did not support the hypothesis that a component of the beneficial metabolic effects of our inhibitors occurred via a CNS-mediated mechanism and led us to consider whether, in fact, the compound may be acting via other off-target mechanisms. To test this, we generated and phenotyped mice with a global knockout of 11β-HSD1 (GKO). These mice had reduced levels of 11β-HSD1 mRNA expression (<97%) (Figure 3A) and enzyme activity (<92%; at the limit of detection) (Figure 3B). Global deletion of 11β-HSD1 did not cause a rebound increase in circulating corticosterone from the adrenal gland at either the peak or nadir (Figure 3D). The effect of 11β-HSD1 inhibitors was then tested in this model. With this approach, any residual effects of 11β-HSD1 inhibition could be attributed to non-11β-HSD1–mediated or off-target actions.

GKO mice did not counteract the body weight gain when fed the HFD and were essentially similar to WT controls (Figure 3D). This lack of effect of whole-body 11β-HSD1 deletion was seen in 5 separate studies. In addition, there were no differences in epididymal, mesenteric, or inguinal fat pad weights (Figure 3, E–G) and no change in percentage body fat (Figure 3H). The dyslipidemia in HFD-fed mice was not improved in GKO mice in that NEFA (Figure 3I) and triglyceride (Figure 3J) levels were similar between GKO and WT mice. There was a marginal, but significant, increase in food intake in the GKO mice (∼6%) compared with that in the WT controls (Figure 3K). In addition, there was no difference between WT and GKO mice in terms of glucose tolerance (Figure 3L) or insulin sensitivity (Figure 3M) during an OGTT. The stress involved in an OGTT may increase corticosterone and therefore increase glucose levels; however, in studies performed by our group in the absence of glucose, multiple repeated blood samples taken over 90 minutes did not increase glucose levels (data not shown).

### Body weight loss produced by 11β-HSD1 inhibitors is predominantly off-target

The lack of metabolic improvement in GKO mice coupled with the requirement for very high concentrations of inhibitor to improve metabolic parameters in C57BL/6J animals further supported the idea that most of the effects seen with compound C were through engagement with another unknown target. To address the possibility of off-target effects, we administered compound C to our GKO mouse strain that had been fed the HFD.

Obese WT and GKO mice were administered compound C (100 mg/kg/d) in their diets for 25 days. At this dose, the compound reduced 11β-HSD1 enzyme activity in WT mice to a level similar to that seen in the GKO mice (Supplemental Figure 3A). Free plasma and brain levels of compound were similar between WT and GKO mice (Supplemental Figure 3, B and C).

**Figure 4.**
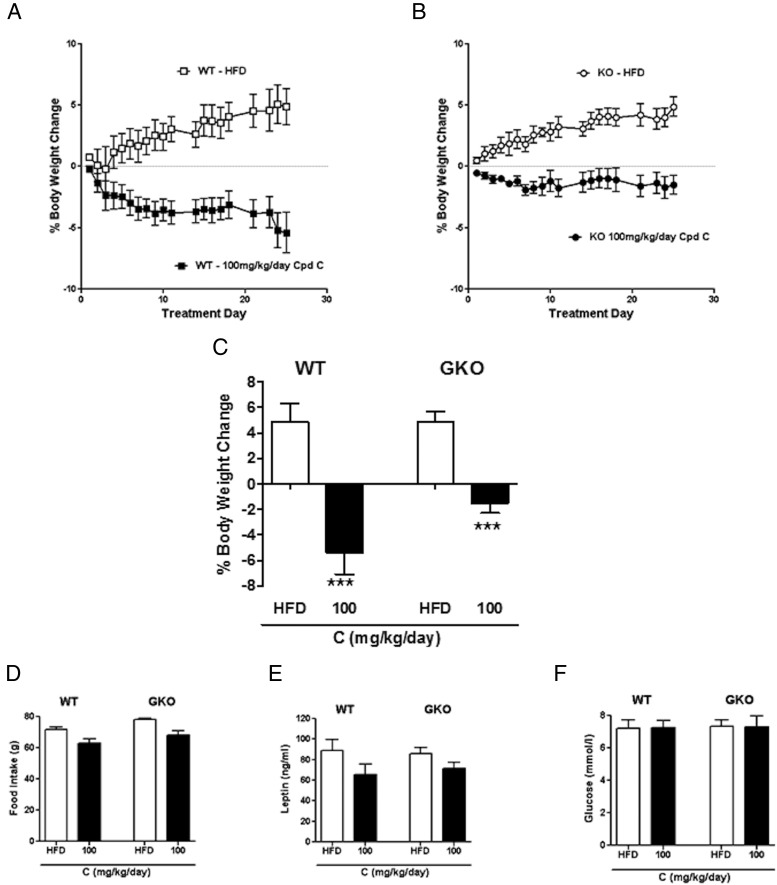
Positive metabolic effects of the 11β-HSD1 inhibitor, compound C, appear to be “off target.” A, Body weight change in WT mice with compound C treatment. □, vehicle-treated WT; ■, 100 mg/kg/d compound C–treated WT. B, Body weight change in GKO mice with compound C treatment. ○, vehicle-treated GKO; ●, 100 mg/kg/d compound C–treated GKO. C, Percentage body weight change on day 25 of treatment. D, Cumulative food intake during the 25-day treatment. E, Fed leptin levels in terminal plasma taken on treatment day 25. F, Fasting glucose levels on treatment day 20. Data are expressed as means ± SEM; n = 5 to 7 mice per group. ***, *P* < .001 vs HFD-fed mice.

WT mice fed the HFD and given 100 mg/kg/d compound C had approximately 10% lower body weights than controls by the end of the treatment period (Figure 4A). However, most of this observed weight loss was also seen in mice in which 11β-HSD1 had been deleted globally (Figure 4B). This finding suggests that much of the body weight loss is off-target (Figure 4C), although analysis by two-way ANOVA did indicate an interaction between groups, making the data more difficult to interpret, so we cannot discount the possibility that there might be a small effect of 11β-HSD1 inhibition. There was also a trend toward a reduction in food intake (Figure 4D) and leptin levels (Figure 4E) in both genotypes treated with inhibitor, but this was not significant nor was there any effect on fasting glucose in this study (Figure 4F).

**Figure 5.**
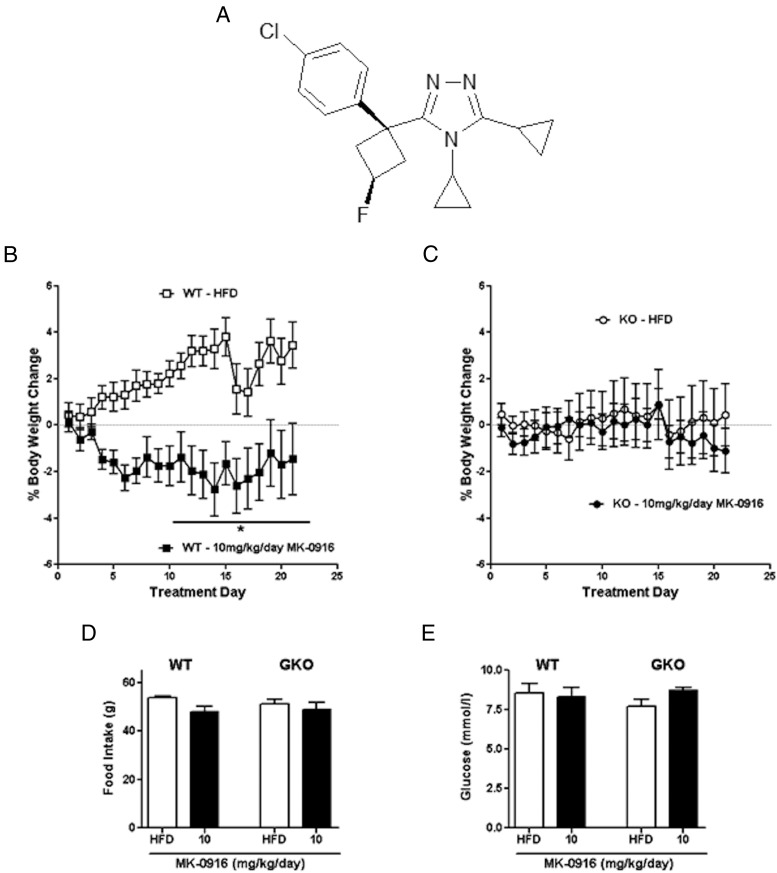
Body weight and food intake effects of a lower dose of the 11β-HSD1 inhibitor, MK-0916, are “on target.” A, Structure of MK-0916. B and C, Body weight change in WT (B) and GKO (C) mice with 10 mg/kg/d MK-0916 treatment. □, vehicle-treated WT; ■, 10 mg/kg/d MK-0916–treated WT; ○, vehicle-treated GKO; ●, 10 mg/kg/d MK-0916–treated GKO. D, Cumulative food intake during the 22-day treatment. E, Fasting glucose levels on treatment day 16. Data are expressed as means ± SEM; n = 5 to 8. *, *P* < .05 vs vehicle treatment for that genotype.

The effects seen with compound C in GKO mice may be specific to this compound or generic to 11β-HSD1 inhibitors. To test this, we administered a compound from a distinct series of chemicals (MK-0916 [23]), which inhibited the enzyme in vitro, and examined its metabolic effects in HFD-fed WT and GKO mice. MK-0916 has been used to treat patients in clinical trials (19, 31). It inhibits peripheral 11β-HSD1 and can also access the CNS at high doses. It was synthesized based on the patent literature (Figure 5A).

MK-0916 (10 mg/kg/d) given to mice fed the HFD reduced enzyme activity in the liver by >90% in WT mice (Supplemental Figure 4A). Free concentrations of the compound were found at similar levels in WT and GKO mice both in plasma (Supplemental Figure 4B) and in brain (Supplemental Figure 4C). Over the 21-day period of treatment with MK-0916, mice lost more weight than their HFD-fed controls (two-way ANOVA) (Figure 5B). This weight loss did not occur in GKO mice (Figure 5C), indicating that it occurred via an 11β-HSD1–specific mechanism. At this dose of compound, no reduction in food intake (Figure 5D) or fed glucose (Figure 5E) was observed in either genotype.

**Figure 6.**
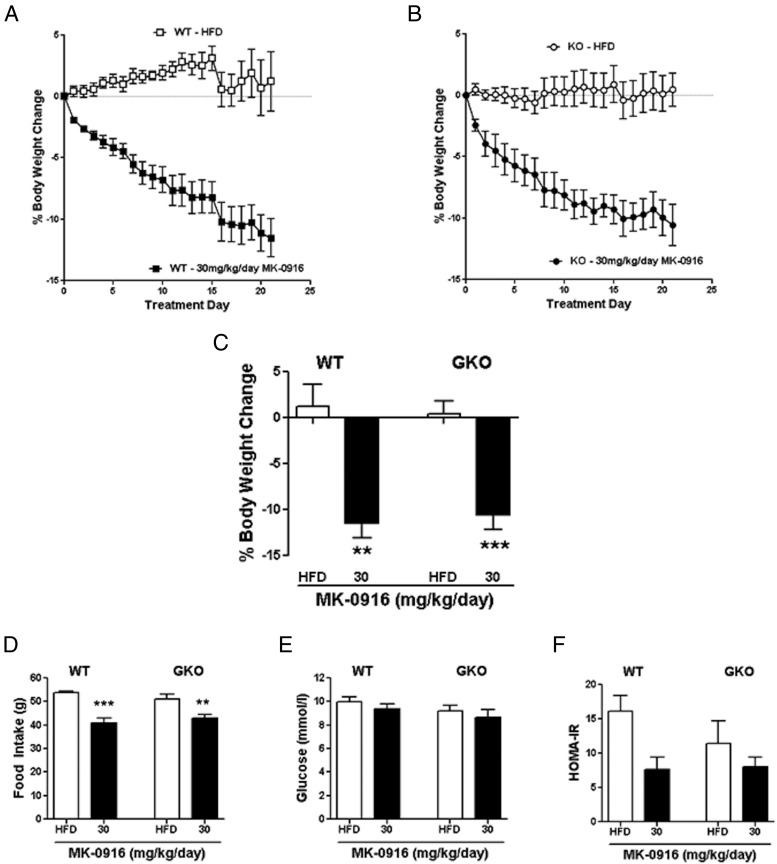
Body weight and food intake effects of a higher dose of the 11β-HSD1 inhibitor, MK-0916, are “off target.” A and B, Body weight change in WT (A) and GKO (B) mice with 30 mg/kg/d MK-0916 treatment. □, vehicle-treated WT; ■, 30 mg/kg/d MK-0916–treated WT; ○, vehicle-treated GKO; ●, 30 mg/kg/d MK-0916–treated GKO. C, Percentage body weight change on day 21. D, Cumulative food intake during the 22-day treatment. E, Fasting glucose levels on treatment day 16. F, Insulin resistance as measured by HOMA-IR at treatment day 16. Data are expressed as means ± SEM; n = 6 to 8. **, *P* < .01; ***, *P* < .001 vs vehicle treatment for that genotype.

Because there was a relatively small weight loss with the 10 mg/kg/d dose of MK-0916, we administered a higher dose of this compound to both WT and GKO mice to get a more clinically relevant body weight loss. Again at this dose, 11β-HSD1 was inhibited by >90% (Supplemental Figure 4D), and free compound levels did not differ between the WT and GKO mice in plasma (Supplemental Figure 4E) and brain (Supplemental Figure 4F). With this higher dose of inhibitor, HFD-fed WT mice lost approximately 12% of their body weight (Figure 6, A and C). A similar magnitude of body weight loss was also observed in GKO mice (Figure 6, B and C), indicating that the body weight loss was off-target. Treatment with MK-0916 caused a reduction in food intake in both WT and GKO mice (Figure 6D), indicating that this was also an off-target effect. No decrease in fed glucose levels (Figure 6E) was observed in either WT or GKO mice. There was a trend toward an improvement in insulin sensitivity, as measured by homeostasis model assessment of insulin resistance (HOMA-IR) in both the WT and the GKO mice treated with MK-0916. The magnitude of improvement in insulin sensitivity appeared to be greater in the WT mice, but results in neither genotype reached significance (Figure 6F).

## Discussion

Inhibition of 11β-HSD1 has been targeted by many pharmaceutical companies as a promising new therapy to treat type 2 diabetes and obesity, primarily because of the anticipated effects in lowering glucose and reducing body weight in obese individuals. However, despite numerous published preclinical studies over the last decade and many compounds entering clinical development, no 11β-HSD1 inhibitor has made it to phase III clinical trials. With the high levels of attrition of phase I/II development compounds, it is not easy to determine whether the lack of success is due to lack of effectiveness at the target or to the individual compounds. For 11β-HSD1, although the original studies with knockout models were encouraging, the studies presented here and more recent data from other groups (32, 33) suggest that there is little improvement in the metabolic phenotype in the absence of 11β-HSD1. In parallel, our studies with 11β-HSD1 inhibitors have produced profiles similar to those of other preclinical studies in HFD-fed C57BL/6 mice in that high doses of inhibitors are needed to successfully reduce blood glucose and body weight. We have addressed this disconnect directly in our present studies, by examining the effects of 11β-HSD1 inhibition in mice lacking this enzyme and shown that although there are some small improvements in metabolic parameters with low doses of compounds, the effects seen with high doses of 2 inhibitors are largely due to off-target mechanisms. We therefore believe that it is of importance to reconsider the true potential of 11β-HSD1 inhibition in the development of novel therapies for treatment of type 2 diabetes and obesity.

A conundrum appears to exist in studies with 11β-HSD1 inhibitors in which low concentrations of compounds maximally inhibited the enzyme, but higher concentrations are needed to have more marked pharmacodynamic effects. In a phase IIa study in patients with type 2 diabetes, MK-0916 (a compound we studied here) failed to lower fasting plasma glucose even with approximately 85% enzyme inhibition in liver but did deliver a modest degree of weight loss (19). Notably, this profile was very similar to that observed with the low dose of MK-0916 in the current preclinical study, which delivered its modest weight loss effects through on-target mechanisms. In a phase IIb clinical trial in type 2 diabetic patients, INCB13739 produced lowering of hemoglobin_A1c_, fasting plasma glucose, and HOMA-IR as well as a modest decrease in body weight, but only at higher doses (≥100 mg). Interestingly, a 50-mg dose of inhibitor already reached 90% inhibition of 11β-HSD1 in the liver (18, 34, 35) but was without effects on hemoglobin_A1c_ or body weight. Thus, clinical data indicate that only very high doses of inhibitors, giving very high levels of enzyme inhibition, are significantly efficacious.

We demonstrated that although inhibition of 11β-HSD1 with small molecule inhibitors is very effective, our selected compound (compound C) at the lower dose (50 mg/kg/d) was not able to produce a relevant improvement in metabolic phenotype. As a caveat, HFD feeding in mice decreases the levels of 11β-HSD1 in adipose tissue (36), and therefore it may be more difficult to demonstrate inhibition of the enzyme. Nevertheless, a 4-fold increase in the dose of compound C (200 mg/kg/d) also resulted in >90% inhibition of the enzyme, but, in addition, gave much larger reductions in body weight, food intake, and fasting glucose levels. The need for very high doses of inhibitor is not restricted to our studies as noted above. This led us to consider that exposures of peripheral tissues to the drug were not the major determinant of efficacy and achieving high CNS drug exposures might be required.

It was apparent that compound C was able to penetrate the brain, because at the high dose the CNS levels reached values greater than those required to inhibit enzyme activity. Furthermore, expression of 11β-HSD1 has been localized to many regions of the brain, one of which is the arcuate nucleus of the hypothalamus (10, 37, 38), an area important for the control of both food intake and glucose homeostasis. Within the CNS, glucocorticoids have been shown to stimulate appetite (39, 40) and to induce hepatic insulin resistance (27). This finding suggested to us that decreasing active glucocorticoids within the CNS might produce beneficial effects in terms of reducing food intake and improving glucose homeostasis. Our generation and phenotyping of mice lacking 11β-HSD1 specifically within the CNS has shown that these animals are almost indistinguishable from their Nestin-*Cre* controls, indicating that expression of this enzyme within the brain contributes minimally to the regulation of body weight and glucose metabolism. However, the BKO mice did have increased food intake compared with the Nestin-*Cre* control mice, but both these genotypes have significantly lower food intake than 11β-HSD1 floxed or C57BL/6J control mice, and therefore the increased food intake in BKO mice, compared with that of WT^nes^ mice, is most likely due to the background strains. Nevertheless, because mice with the Nestin-*Cre* construct have a metabolic phenotype (Supplemental Figure 2 and Refs. 29, 30), we cannot discount the possibility that the presence of Nestin-*Cre* may be masking a subtle lowering of body weight, food intake, and/or glucose levels caused by knockout of 11β-HSD1 in the brain.

The lack of a beneficial metabolic effect when 11β-HSD1 was knocked out in the CNS suggests that the requirement for a high dose of inhibitor to have a metabolic effect is not due to a need to access the brain for efficacy. Our alternative hypothesis was that the greater metabolic effects of high doses of 11β-HSD1 inhibitors could be mediated via non-11β-HSD1, ie, off-target mechanisms. To test this we evaluated the effects of compounds in GKO mice. The GKO mouse we developed did not display metabolic improvements (no effect on body weight gain, adiposity, or insulin sensitivity) and even had increased food intake. In contrast, the original GKO phenotype showed improvements in glucose tolerance and reduced body weight gain with the HFD (11), and this was maintained when the genotype was backcrossed onto C57BL/6 mice. These mice were shown to have increased core body temperature, which was thought to be responsible, at least in part, for the reduced body weight (41). This may explain the increased food intake without alterations in body weight in the present study. However, more recently, there has been some disparity between published studies, with some not finding the same degree of metabolic protection of GKO (32, 33), which is in accordance with our own data presented here, and other recent studies showing some small degree of metabolic improvement (42). Why differences are seen is not clear; however, the reason may be small differences in strain background or housing conditions.

Our findings from the GKO studies indicate that with a low dose of 11β-HSD1 inhibitor there are some 11β-HSD1-dependent decreases in body weight, as seen with MK-0916. These reductions in body weight are not likely to be relevant for a novel type 2 diabetes and obesity therapy, the target for many of these inhibitors entering clinical trials. However, at higher doses, the 11β-HSD1 inhibitors gave greater levels of efficacy. Therefore, in this, and possibly other, preclinical mouse studies, these larger improvements are likely to be partially due to off-target mechanisms. It is, however, unexpected that 2 compounds with completely different chemistry would give similar off-target effects. A potential explanation for this may be that high doses of compounds induce nausea; however, the mice were closely monitored for symptoms and showed no ill effects while receiving the treatment.

There has been a huge drive to develop pharmacological approaches for inhibiting 11β-HSD1 as a new therapy for type 2 diabetes and obesity. However, to date, the development of all these inhibitors has been halted before phase III clinical trials. There may be a number of reasons for this, including potential side effects in relation to perturbation of the hypothalamic-pituitary-adrenal axis (43). However, the data reported herein demonstrate that selective inhibition of 11β-HSD1 can only deliver small improvements in glycemic control and body weight and that the larger improvements observed in preclinical trials are likely to be off-target. Indeed, they support the hypothesis that only limited effects are likely clinically, suggesting that the clinical effects observed to date may be the best that can be expected from selective inhibition of 11β-HSD1. Recent preclinical evidence suggesting a role for 11β-HSD1 in the development of atherosclerotic lesions (44, 45) may open up additional aspects of cardiovascular disease that may be targeted by 11β-HSD1 inhibition. Nevertheless, it appears that at present, the selective inhibition of 11β-HSD1 will only lead to small improvements in glucose control and obesity and therefore cannot be considered to be a strong therapeutic target for producing a step-change treatment for obesity or type 2 diabetes.
